# Implications of clinical simulation in motivation for learning: scoping review

**DOI:** 10.31744/einstein_journal/2024RW0792

**Published:** 2024-04-10

**Authors:** Barbara Casarin Henrique-Sanches, Dario Cecilio-Fernandes, Raphael Raniere de Oliveira Costa, Rodrigo Guimarães dos Santos Almeida, Federico Ferrero Etchegoyen, Alessandra Mazzo

**Affiliations:** 1 Universidade de São Paulo Escola de Enfermagem de Ribeirão Preto Ribeirão Preto SP Brazil Escola de Enfermagem de Ribeirão Preto, Universidade de São Paulo, Ribeirão Preto, SP, Brazil.; 2 Universidade Estadual de Campinas Campinas SP Brazil Universidade Estadual de Campinas, Campinas, SP, Brazil.; 3 Universidade Federal do Rio Grande do Norte Natal RN Brazil Universidade Federal do Rio Grande do Norte, Natal, RN, Brazil.; 4 Universidade Federal de Mato Grosso do Sul Campo Grande MS Brazil Universidade Federal de Mato Grosso do Sul, Campo Grande, MS, Brazil.; 5 Universidade Nacional de La Plata Faculdade de Ciências Médicas Buenos Aires Argentina Universidade Nacional de La Plata, Faculdade de Ciências Médicas, Buenos Aires, Argentina.; 6 Universidade de São Paulo Bauru SP Brazil Universidade de São Paulo, Bauru, SP, Brazil.

**Keywords:** Motivation, Simulation training, Education, nursing, Education, medical, Learning, Learning health system

## Abstract

**Objective:**

To identify, synthesize, and analyze the scientific knowledge produced regarding the implications of using clinical simulation for undergraduate nursing or medical students' motivation for learning.

**Methods:**

The search for articles was conducted between July 28 and August 3, 2022, on the PubMed/MEDLINE, Scopus, Web of Science, and SciELO databases. The following was used for the search: P - undergraduate students attending Nursing or Medicine courses; C - motivation for learning, and C - skills and clinical simulation laboratory. The following research question guided the study: “What are the implications of clinical simulation on the motivation for learning of undergraduate students of nursing and medicine?” Of the 1,783 articles found, 13 were included in the sample for analysis. All stages of the selection process were carried out by two independent evaluators. The results were presented as charts and a discursive report.

**Results:**

The studies analyzed indicated the beneficial effects of clinical simulation on students' motivation, in addition to other gains such as competencies, technical and non-technical skills, knowledge, belonging, autonomy, clinical judgment, critical and reflective thinking, self-efficacy and decreased anxiety, self-management, and improvements in learning and learning climate.

**Conclusion:**

Clinical simulation provides a positive learning environment favorable to the development of technical and interpersonal skills and competencies, and raising the level of motivational qualities.

## INTRODUCTION

In education, motivation plays a decisive role in students' level of involvement in the acquisition of knowledge, skills, and atitudes.^(1)^ When motivated, students become actively involved, demonstrate persistence and engagement in academic activities and enthusiasm during the execution of tasks, and apply their energy and efforts to focus on their learning.^(2)^

Although there are several theories about motivation, self-determination theory (SDT) has gained prominence in the educational context.^(1)^ Self-determination theory postulates that socio-contextual conditions contribute or inhibit processes of self-motivation and healthy psychological development.^(1,3,4)^

In addition to different levels of motivation, SDT proposes that there are three basic psychological needs-autonomy, competence, and belonging-which collaborate to promote intrinsic motivation and motivational quality.^(3-5)^ Considered the central construct in SDT, autonomy refers to a desire in relation to a goal and the need to feel in control of one's own actions with the absence of external impositions. Competence refers to the feeling related to the sense of confidence and efficacy in the activity. This is developed in interactions with the environment, in which experiencing opportunities for practice are experienced, and in expressing their knowledge and technical and attitudinal skills. Finally, belonging provides a sense of involvement with the other and security. It is the feeling of connection, of relationship, and of perceiving oneself as part of a group.^(4)^

The learning process comprises internal and external motivational elements and commands a challenging environment for students. However, teaching activities developed to stimulate motivation must concretely encourage learning through active methods. For this to be effective, it must involve students through social interaction, and create an environment in which peers and teachers can bond in the process of constructing and reconstructing their knowledge.^(6)^ In this sense, factors related to methodology and teaching strategies can trigger components that increase or decrease intrinsic motivation, the level of internalization of extrinsic motivation, and the well-being achieved through satisfying basic psychological needs.^(1,3,4)^ Among the various active instructional methods that have proven effective for this purpose, clinical simulation stands out among health professionals.^(7,8)^

Clinical simulation is a technique that creates situations to allow experiences that resemble circumstances experienced in reality in an interactive manner^(9)^ and in a controlled and risk-free environment, especially for the patient. It also enables the integration of knowledge, attitudes, skills, and individual or collective decision making.^(10)^

This study seeks to better understand how this process occurs and the components that can enhance it.

## OBJECTIVE

This study aimed to identify, synthesize, and analyze the scientific knowledge on the implications of using clinical simulation in motivation for learning in undergraduate nursing or medical students.

## METHODS

### Project

This scoping review follows the scoping review strategy proposed by the Joanna Briggs Institute.^(11)^ This review was structured according to the following stages: the question guiding the review and objective were formulated, the search strategy was elaborated, the databases were searched, articles were selected based on reading their titles and abstracts, scientific articles were selected based on their full-text reading, the results were summarized, and the results were presented and discussed.

To formulate the research guiding question and search strategy, the population, concept, and context (PCC) strategy was used.^(11)^ Thus, P was defined as “undergraduate students in nursing or medicine,” C as “motivation for learning,” and C as “skills and clinical simulation laboratory.” Based on this definition, the following guiding question was formulated: “What are the implications of clinical simulation on the motivation for learning of undergraduate students of nursing and medicine?”

The inclusion criteria were articles that contained the three PCC elements; which answered the research question; were written in English, Portuguese, or Spanish; in any period. Articles written in other languages and that did not answer the guiding question were excluded.

### Search strategy

The search for articles was conducted between July 28 and August 3, 2022, on the following databases: the National Library of Medicine (PubMed/MEDLINE), Scopus, Web of Science, and Scientific Electronic Library Online (SciELO).

For the search, Descriptors in Health Sciences (DECS - *Descritores em Ciências da Saúde*) and Medical Subject Headings (MeSH) were used for each item of the strategy and its related terms. To combine descriptors, the AND, OR, and NOT Boolean operators were used. Thus, the following descriptors were used for Population (P): (“professional education” OR “nursing education” OR “nursing diploma programme” OR “medical education” OR “undergraduate medical “ OR “health occupations students” OR “medical students” OR “nursing students”). For Concept (C), we used: (“learning”) AND (“motivate” OR ‘motivation” OR “learning motivation”), and for Context (C): (“simulation training” OR “interactive learning” OR “high fidelity simulation training” OR “patient simulation” OR “simulation” OR “simulator”).

The PRISMA Extension for Scoping Reviews (PRISMA-ScR) guidelines and checklist was followed.^(12)^

### Search results

The search of the 4 databases resulted in 1,783 articles: 853 (48.0%) in Web of Science, 592 (33.0%) in PubMed, 307 (17.0%) in Scopus, and 31 (2.0%) in SciELO. With the support of Rayyan^®^^(13)^ software, 554 duplicate studies were excluded. After carefully reading the titles and abstracts of the remaining 1,229 articles, 76 were selected for a full-text reading. Six references were also found in the Gray Literature and added to the selection process, totaling 82 articles for the full-text reading.

**Figure 1:**
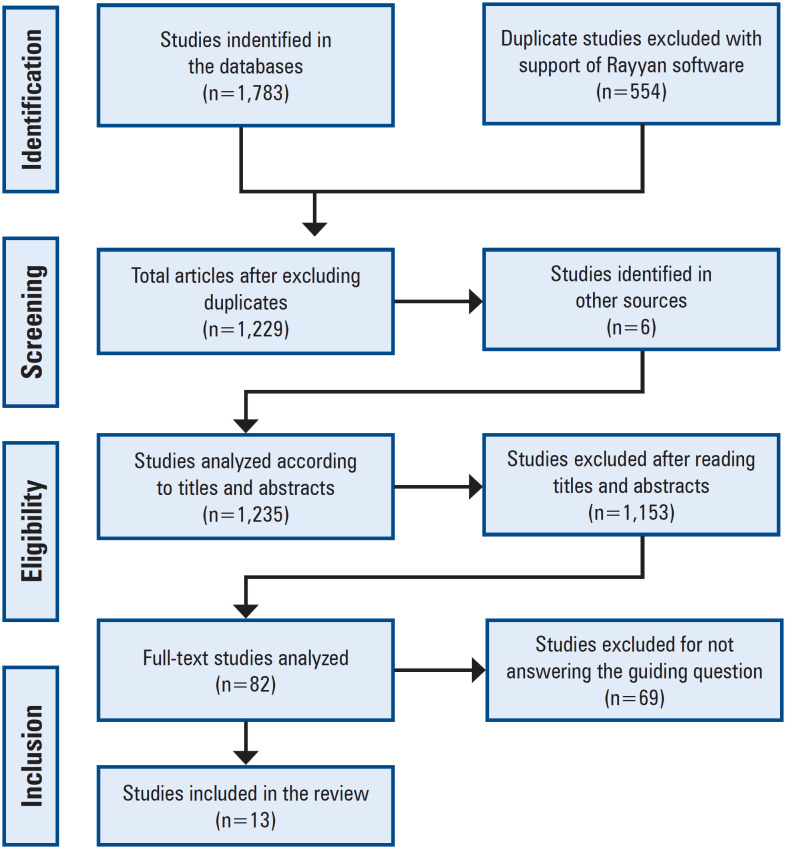
Study selection process

After reading the 82 selected articles, 13 were included in the study sample because they met the established criteria. All stages of the selection process were conducted by two independent evaluators according to the JBI criteria, and are shown in figure 1.

### Quality assessment

The quality or risk of bias of the studies were not assessed. This approach is consistent with the guidance of the scoping review pioneers.^(14,15)^

For analysis purposes, the articles were numbered from 1 to 13 and called “Study” (S). The results were presented in the form of charts and a discursive report.

## RESULTS

The 13 articles included^(16-28)^ in this study were published from 2015 to 2022, all in English. Regarding their origin, with seven (54%) were from Europe, four (31.0%) from Asia, and two (15.0%) from North America.

Regarding the populations under study, eight articles^(16,18-23,28)^ (61.5%) were carried out with nursing students and five articles^(17,24-27)^ (38.5%) with medical students. For methodological design, 11 studies^(16-18,20-24,26-28)^ (84.6%) are quantitative, 1 (7.7%) is qualitative, and another 1 (7.7%) is a systematic review.

The Situational Motivation Scale^(29)^ and Motivated Strategies for Learning Questionnaire^(30)^ instruments were predominantly used. The Situational Motivation Scale^(29)^ aims to assess the motivation to perform a specific task, measuring an individual's motivational quality. The Motivated Strategies for Learning Questionnaire^(30)^ evaluates undergraduate students' types of self-regulated learning strategies and academic motivational orientation. These instruments are widely used in the literature to assess the motivation for learning at different educational levels and contexts, especially by scholars of educational psychology.^(16,18,21,22,24,26-28)^

Table 1 shows the studies analyzed according to authorship, year of publication, objectives, materials and methods, data collection instruments, strategy employed in the clinical simulation, population, sample size, and country of origin.

**Table 1:** Studies analyzed, authorship, year of publication, objectives, methodology, data collection instruments, simulation used, population, sample size, and country of origin

Authorship	Objectives	Materials and methods	Data collection instruments	Characterization of the clinical simulation used	Country, population, and sample size
Roh et al.^(16)^	To assess motivation and life skills (communication, problem-solving, and self-direction) before and after interventions with clinical simulation and problem-based learning	Quasi-experimental, pre- and post-intervention (pre-test)	Motivated Strategies for Learning Questionnaire (MSLQ)^(30)^	A simulated urgency and emergency scenario with a high-fidelity simulator	South Korea, 83 second-year undergraduate nursing students
Thoma et al.^(17)^	To compare intrinsic motivation related to participation in curricular clinical simulation sessions with predetermined learning objectives and extracurricular clinical simulation sessions with learning objectives determined by the students	Non-randomized crossover study	Intrinsic Motivation Inventory^(31)^	A simulated urgency and emergency scenario with a high-fidelity simulator	United States, 22 first-year undergraduate medical students
Fawaz et al.^(18)^	To evaluate the impact of clinical simulation on clinical judgment and students' motivation	Multicenter, post-test, quasi-experimental, case-control study	Lasater Clinical Judgment Rubric;^(32)^ Motivated Strategies for Learning^(30)^	A simulated urgency and emergency scenario with a high-fidelity simulator	Lebanon, 56 first-year undergraduate nursing students
Jeppesen et al.^(19)^	To explore the literature on the connection between teaching and learning strategies for nursing students to clarify which methods provide optimal learning outcomes	A systematic literature review that included quantitative and qualitative studies	Does not apply	Does not apply	Denmark, Nursing.
Park et al.^(20)^	To evaluate the effects of clinical simulation on critical thinking, skills development, self-efficacy, and motivation for learning	Quasi-experimental, pre- and post-test study, with no control group	The Self-Efficacy Scale;^(33)^ Instructional Materials Motivation Scale;^(34)^ and Institutional protocol for testing individual skills	A simulated urgency and emergency scenario with a high-fidelity simulator	South Korea, 69 fourth-year undergraduate nursing students
Sarikoc et al.^(21)^	To assess the impact of using standardized patients in simulated psychiatric cases on students' levels of motivation and perceived learning, when compared to traditional teaching	Quantitative, randomized and controlled study, pre- and post-test	Motivated Strategies for Learning Questionnaire (MSLQ);^(30)^ Perceived Learning Scale;^(35)^ and Educational method evaluation^(21)^	A simulated scenario with simulated patients	Turkey, 86 third-year undergraduate nursing students
Walters et al.^(22)^	To evaluate knowledge retention and the perception of the learning climate (autonomy, competencies, relationships, and motivation) after simulated practices	Longitudinal case-control study	Learning Climate Questionnaire;^(36)^ Basic Psychological Needs at Work Scale.^(37)^ The Situational Motivation Scale^(29)^	Hybrid simulation using low-fidelity simulators and simulated patients	United States, 199 third-year undergraduate nursing students
Guerrero-Martínez et al.^(23)^	To evaluate students' learning in a simulated emergency scenario	Pre- and post-test quasi-experimental study	Adapted questionnaire called “*Encuesta de calidad y satisfacción de simulación clínica en estudiantes de enfermería*”^(38)^	Hybrid Multiple Casualty Incident simulation with standardized patients and low- and medium-fidelity simulators	Spain, 47 fourth-year undergraduate nursing students
Schulte-Uentrop et al.^(24)^	To analyze the level of intrinsic motivation in the performance of non-technical skills	Prospective and cross-sectional cohort study	The Situational Motivation Scale (SIMS)^(29)^ Non-technical skills classification tool^(24)^	A simulated urgency and emergency scenario with a high-fidelity simulator	Germany, 449 students from first to fourth year of the medicine undergraduate course
Lee et al.^(25)^	To explore, categorize, and articulate the factors that interfere with the motivation for learning during simulated clinical practice	Case study with qualitative analysis and inductive thematic approach using the *Dedoose* software	Debriefing session with semi-structured questions	Clinical simulation of patient consultation standardized by students	United Kingdom, 17 third-year undergraduate medical students
Mool-Khosrawi et al.^(26)^	To identify and compare students' situational motivation in bedside teaching and clinical simulation	Prospective cohort, pre- and post-test, descriptive, with no Control Group	The Situational Motivation Scale^(29)^	A simulated urgency and emergency scenario with a high-fidelity simulator	Germany, 145 third-year undergraduate medical students
Mool-Khosrawi et al.^(27)^	To assess and compare students' motivational levels before and after clinical simulations and classic seminars	Pre- and post-test quasi-experimental and cross-sectional study	The Situational Motivation Scale^(29)^	A simulated urgency and emergency scenario with a high-fidelity simulator	Germany, 164 third-year undergraduate medical students
Arizo-Luque et al.^(28)^	To assess motivation for learning and critical thinking before and after self-directed training based on clinical simulation	Multicenter study, pre- and post-tests, cross-sectional, descriptive, quantitative, with no Control Group	Motivated Strategies for Learning Questionnaire (MSLQ-44)^(30)^ and N-CT-4 Practice (Nursing Critical Thinking in Practice Questionnaire^(39)^	Simulated emergency scenarios and non-technical skills, use of high-fidelity simulators or standardized patient	Spain, 77 third-year undergraduate nursing students

Among the studies analyzed, there were several gains for the students along with motivation. Regarding the implications of clinical simulation on students' motivation for learning, we found that most studies^(16-23,25,28)^ showed a positive relationship between these two components. In addition to the beneficial effects on students' motivation and motivational qualities, simulation fostered numerous gains in terms of competencies,^(21,22)^ technical^(23,26,28)^ and non-technical skills,^(16,28)^ knowledge,^(22,28)^ belonging,^(17,28)^ autonomy,^(17)^ clinical judgment,^(18,28)^ critical and reflective thinking,^(19,20)^ self-efficacy and self-confidence,^(20,21,23,27,28)^ decreased anxiety,^(21)^ self-management^(28)^ improvements in the learning environment,^(22)^ satisfaction,^(23)^ and learning in general terms. These, together or individually, are all directly related to motivation.^(4)^ Table 2 presents the main results and conclusions in this regard.

**Table 2:** Main results and conclusions of the studies

Authorship	Main results and conclusions of the studies
Roh et al.^(16)^	Motivation for learning and problem-solving increased from the pre-test and post-PBL compared to after the clinical simulation. Self-directed communication and learning increased from the pre- to post-test after the clinical simulation. Integrated with the clinical simulation, the PBL method significantly improved students' motivation and life skills.
Thoma et al.^(17)^	Extracurricular clinical simulation sessions with learning objectives determined by the students increased their perceived autonomy, although there was no difference in intrinsic motivation, competence, and belonging when compared to clinical simulation sessions devised by the facilitators. However, the level of intrinsic motivation was very high in both, indicating that clinical simulation is a motivating method independent of autonomy in choosing learning objectives.
Fawaz et al.^(18)^	The clinical simulation significantly improved students' clinical judgment and motivation.
Jeppesen et al.^(19)^	The skills and clinical simulation laboratories provided a positive and motivating learning environment, and developed students' critical-reflective thinking.
Park et al.^(20)^	The simulated practice increased critical thinking, self-efficacy, and motivation for learning.
Sarikoc et al.^(21)^	The students who underwent simulated practices showed increased motivation, perceived learning, and self-confidence, as well as decreased anxiety when caring for real patients.
Walters et al.^(22)^	The students who underwent simulated practices had more knowledge and a better perception of the learning environment (autonomy, competencies, relationships, and motivation).
Guerrero-Martínez et al.^(23)^	Clinical simulation in the emergency setting increased satisfaction, confidence, motivation, perception of decision-making, and technical skills.
Schulte-Uentrop et al.^(24)^	High autonomous motivation levels were observed in all students, but this did not correlate with the better performance of non-technical skills in simulation-based emergency training.
Lee et al.^(25)^	Integrating simulated surgical clinics into the curriculum eased skills acquisition through active learning. Enjoyment of the simulated session was identified as an intrinsic motivator. It also enabled students to recognize the importance of preparing themselves for clinical practice, rather than focusing on theoretical evaluations.
Mool-Khosrawi et al.^(26)^	High autonomous motivation levels (intrinsic and identified regulation) and low controlled motivation levels (introjected and external) and demotivation were reported pre- and post-test during the simulated practices and bedside teaching. Clinical simulation had no significant effect on the different motivational qualities. Bedside teaching significantly decreased extrinsic motivation and identified motivation.
Mool-Khosrawi et al.^(27)^	The different motivational qualities and motivation levels did not differ significantly when comparing simulated practices to traditional seminars.
Arizo-Luque et al.^(28)^	After the simulated clinical practices, the students improved their general levels of motivation (self-efficacy, learning strategy, self-regulation) and critical thinking (personal characteristics, intellectual, cognitive and interpersonal skills, self-management, and technical skills).

## DISCUSSION

The efficacy of the learning process is multifactorial. Herein, motivation plays a fundamental role in determining students' level of involvement and efforts in knowledge construction, directing behaviors toward a goal, and in the variability and quality of the actions applied in teaching situations.

In this review, we mapping the knowledge produced on the implications of clinical simulation on nursing and medical students' motivation. This showed that studies on this subject began in 2015. Likely, this is due to the increase in research on clinical simulation during this period and to improvements in theoretical frameworks and research methods, which occurred mainly among North American, European, and Asian researchers. German researchers are the most published on this topic. 

Regarding the simulated clinical activities developed in these studies to evaluate motivation, emergency medicine scenarios using high-fidelity simulators to assess technical and non-technical skills dominated. However, the use of simulated patients and hybrid simulations was also observed, which combine simulated patients and use low- and medium-fidelity simulators in the same scenario. In the simulation, the resources (human, physical, and material) and activity to be developed (skills training and/or development of the scenario) must be in accordance with the learning objectives outlined. These also differ in the degree of task complexity.^(40)^ Studies that employ emergency medicine scenarios are common, which can be attributed to the relevance of the topic, the current incorporation of this content in health courses, and the fact that this knowledge area has elaborated and frequently revised action protocols, which eases and standardizes the evaluation process.

Professionals in training aim to acquire competence in terms of theoretical knowledge and technical and non-technical skills, which will culminate in a feeling of self-confidence and self-efficacy.^(41)^ Self-confidence refers to people's belief in their skills, while self-efficacy is their ability to accomplish a task or achieve a goal.^(42)^ Self-confidence and self-efficacy contribute to a sense of competence, which promotes feelings of autonomy and self-determination that imply the level of motivation. In turn, competence is directly related to motivation. It is part of the triad of basic psychological needs. 

Various authors emphasize the benefits of simulated teaching in the development of hard skills, because through this method, students better learn what to do and how to do it in a safe and risk-free environment. Furthermore, the possibility of unlimited repetitions increases their self-confidence, self-efficacy, and development of the necessary skills. This promotes intrinsic motivation and satisfactory self-regulated motivation levels, as they are related to satisfying the basic psychological need of competence.^(4,23,24,28,43)^

In the sample of studies analyzed here, in terms of motivation,^(16,17)^ soft or interpersonal skills are non-technical and do not depend on abstract reasoning.^(44,45)^ In a simulation, they are developed through communication skills, work organization, teamwork, and critical thinking, among others, which gives the opportunity to analyze problems from multiple perspectives and positively impacts clinical reasoning and decision-making processes.^(41)^ Soft skills involve self-awareness, decision-making and emotional management, critical and reflective thinking, empathy, problem-solving, teamwork, effective communication, self-efficacy, self-confidence, creativity, cooperation, negotiation, self-management, resilience, and respect for diversity.^(45)^ They complement hard skills and allow students to resolutely deal with professional and everyday life challenges. They can be learned and developed through proper training. As such, in terms of health education, interprofessional work, patient safety, and a more humanized perspective as professionals and patients evolve, educational institutions have incorporated soft skills into their curricula.^(44,45,47)^

Clinical skills and clinical judgment are a set of diverse knowledge and practices required for professional practice such as anamnesis, physical examination, diagnostic reasoning, and attitudinal skills like communication and empathy. This learned behavior integrates hard and soft skills, which enhance competence, confidence, and efficacy, and intensifies the motivation for learning.^(18,48)^

The development and debriefing resolution of a simulated clinical scenario reinforces students' autonomy, self-awareness, decision-making, and critical thinking. Here, autonomy and self-direction relate to yet another of the components of the triad of basic psychological needs, which provide increased intrinsic motivation and reflect improved learning quality and positive academic performance.^(4,28,49)^

A study that compared the levels of motivation for learning in clinical simulation and bedside teaching found that the level of autonomous motivation decreased in the post-test in bedside teaching.^(26)^ The fact that students are not familiar with the hospital environment and the team reduces their sense of belonging and increases their fear of making mistakes. This may expose patients to risk, which reduces the feeling of competence and is ultimately reflected in the level of motivation.

Clinical simulation contributes to the sense of belonging, being the last component of basic psychological needs. As students are introduced to theorizing and presented with new elements, they become interested in knowledge, collaboration with peers, commitment, perception of competence and belonging, and continuity in the learning process. Linked to their active role, this will enable them to exercise their autonomy.^(50-51)^

Furthermore, we found that the majority of the studies analyzed are of European origin, which may limit their applicability in other contexts such as in Central and South American countries, where no studies were found. This highlights the need for research that evaluates the motivation for learning via clinical simulation of nursing and medical students in Latin America. 

## CONCLUSION

Based on this review, it is concluded that clinical simulation provides a positive learning environment favorable to the development of technical and interpersonal skills. It also encourages the development of basic psychological needs, as postulated by self-determination theory, through the triad of competence, autonomy, and belonging. When these are achieved and met, intrinsic motivation and the level of internalization of external regulation increases, which raises the level of motivational qualities.
